# Circulating N-Acetylaspartate Levels Associate with Measures of Peripheral and Tissue-Specific Insulin Sensitivity

**DOI:** 10.3390/ijms26115107

**Published:** 2025-05-26

**Authors:** Eleni Rebelos, Miikka-Juhani Honka, Aino Latva-Rasku, Johan Rajander, Paulina Salminen, Ioanna A. Anastasiou, Dimitris Kounatidis, Nikolaos Tentolouris, Beatrice Campi, Angela Dardano, Giuseppe Daniele, Alessandro Saba, Ele Ferrannini, Pirjo Nuutila

**Affiliations:** 1 Turku PET Centre, University of Turku, 20014 Turku, Finland; mjhonk@utu.fi (M.-J.H.); pirjo.nuutila@utu.fi (P.N.); 2 Department of Clinical and Experimental Medicine, University of Pisa, 56126 Pisa, Italy; angela.dardano@unipi.it (A.D.); giuseppe.daniele@unipi.it (G.D.); 3 Diabetes Center, First Department of Propaedeutic and Internal Medicine, Medical School, National and Kapodistrian University of Athens, Laiko General Hospital, 11527 Athens, Greece; anastasiouiwanna@gmail.com (I.A.A.); dimitriskounatidis82@outlook.com (D.K.); ntentolouris@yahoo.gr (N.T.); 4 Turku PET Centre, Accelerator Laboratory, Åbo Akademi University, 20520 Turku, Finland; johan.rajander@abo.fi; 5 Division of Digestive Surgery and Urology, Turku University Hospital, 20520 Turku, Finland; paulina.salminen@tyks.fi; 6 Department of Surgery, University of Turku, 20014 Turku, Finland; 7 Consiglio Nazionale delle Ricerche (CNR), Institute of Clinical Physiology, 56124 Pisa, Italy; bcampi.bc@gmail.com (B.C.); eleferrannini@gmail.com (E.F.); 8 Laboratory of Clinical Pathology, St. Chiara University Hospital, 56100 Pisa, Italy; alessandro.saba@unipi.it; 9 InFLAMES Research Flagship, University of Turku, 20014 Turku, Finland; 10 Department of Endocrinology, Turku University Hospital, 20520 Turku, Finland

**Keywords:** N-acetylaspartate, insulin sensitivity, euglycemic hyperinsulinemic clamp, positron emission tomography

## Abstract

N-acetylaspartate (NAA) is the second most abundant metabolite in the human brain. Quantifiable amounts of NAA are also present in the blood, but its role in the peripheral tissues is largely unknown. First, we determined the acute effects of insulin administration on NAA concentrations; second, we assessed whether circulating NAA levels associate with markers of central and peripheral insulin sensitivity. A total of 24 persons living with obesity and 19 healthy, lean controls, without neurological disorders, underwent a euglycemic hyperinsulinemic clamp combined with fluorodeoxyglucose positron emission tomography ([^18^F]FDG-PET) imaging of the brain, abdomen, and femoral area. Plasma concentrations of NAA were measured at baseline and ~2 h into the clamp using high-performance liquid chromatography coupled with tandem mass spectrometry (HPLC-MS-MS). Glucose uptake (GU) rates were analysed using a fractional uptake rate. Serum acetate levels were also assessed using nuclear magnetic resonance (NMR) metabolomics. From baseline to steady-state, insulin levels increased from a mean level of 66 to 447 pmol/L (*p* < 0.0001). Over this period, circulating NAA concentrations decreased by 5% (*p* = 0.01), similarly in both groups. The change in NAA was inversely related with the change in plasma acetate (*r* = −0.36, *p* = 0.048). Circulating NAA was associated with waist–hip ratio (*rho* = −0.54, *p* = 0.0002), steady-state free fatty acids (*rho* = −0.44, *p* = 0.003), and directly with HDL cholesterol (*rho* = 0.54, *p* = 0.0002), adiponectin (*rho* = 0.48, *p* = 0.003), and whole-body insulin sensitivity (*rho* = 0.34, *p* = 0.03). Circulating NAA was directly related with skeletal muscle (*rho* = 0.42, *p* = 0.01) and visceral adipose tissue GU (*rho* = 0.41, *p* = 0.02). Insulin administration leads to a small decrease in circulating NAA levels, and NAA associates consistently with markers of insulin sensitivity. While plasma NAA may be relevant to aspects of whole-body homeostasis, mechanistic insights are needed.

## 1. Introduction

N-acetylaspartate (NAA) is an acetylated amino acid that represents the second most abundant metabolite in the human brain [1]. The gene *Nat8l* encodes aspartate N-acetyltransferase, the enzyme responsible for the synthesis of NAA from aspartate and acetyl-CoA. NAA is degraded by aspartoacylase (ASPA), yielding aspartate and acetate [2]. Acetyl-CoA can be regenerated from acetate through the action of acetyl-CoA synthetase and subsequently used for energy production and lipid synthesis. In the brain, NAA metabolism is highly compartmentalized: NAA is produced in neurons, which cannot metabolize it. Instead, NAA exits the neurons and is hydrolysed in oligodendrocytes [2].

Although the brain functions of NAA are not fully understood, its measurement through ^1^H-magnetic resonance spectroscopy (^1^H-MRS) is frequently applied in both clinical and experimental settings. Decreased central NAA levels are considered markers of poor neuronal health, as they have been observed in dementia and various neurocognitive disorders [3,4,5,6,7,8,9]. Conversely, excessive brain NAA concentrations are also poorly tolerated. Patients with Canavan disease exhibit significantly elevated brain NAA levels due to a deficiency in ASPA. Thus, the available evidence suggests that brain NAA levels are tightly regulated, as marked changes in its concentration have deleterious effects.

To date, the study of NAA has primarily focused on central nervous system pathology. However, NAA circulates in quantifiable amounts in the blood and is excreted in the urine [10,11]. Moreover, the enzymatic machinery responsible for producing and cleaving NAA is expressed in several peripheral tissues [12], suggesting that NAA may also have a role in peripheral metabolism.

Previously, we conducted a brain imaging study to examine the impact of obesity and insulin resistance on brain metabolism and metabolites [13]. Our findings demonstrated that circulating NAA levels are not associated with central NAA levels [14]. Moreover, using a random subset of The Maastricht Study, we found that circulating NAA does not associate either with cognitive function or with markers of cerebral small vessel disease [14]. To date, the potential effects of circulating NAA in humans remain unclear. In the present study, we aimed to deepen our understanding regarding circulating NAA in humans. Using the data of the same dataset [13,14], we first assessed whether insulin administration affects circulating NAA levels in euglycemic hyperinsulinemic clamp experiments (i.e., steady-state NAA, ssNAA). Second, we examined the associations between circulating NAA measured under fasting and insulin clamp conditions with common metabolites related to whole-body metabolism (e.g., free fatty acids (FFA), lipids) and tissue-specific insulin sensitivity measurements from [^18^F]FDG-PET studies.

## 2. Results

The clinical and metabolic characteristics of the study participants are summarized in Table 1. Individuals living with obesity and lean controls were well matched for sex and age. As expected, there were significant differences in adiposity, inflammatory markers, and insulin sensitivity (indexed by the M value) between the two groups. Renal function, as indexed by the estimated glomerular filtration rate (eGFR, mL/min/1.73 m^2^), was similar between the groups.

### 2.1. Fasting vs. Insulin Clamp Experiments

**Figure 1:**
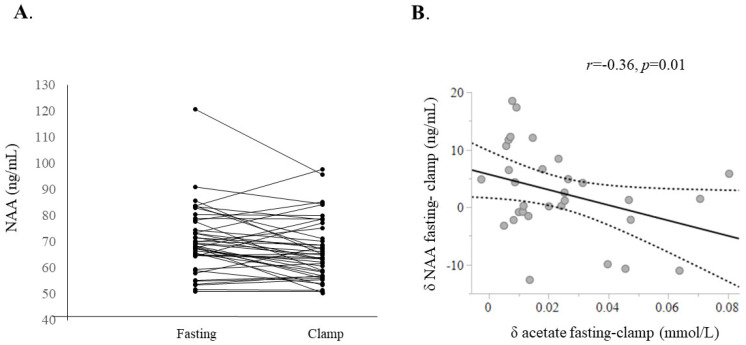
Change in circulating NAA from fasting to the insulin clamp state (**A**). Change in plasma NAA was inversely related to change in acetate levels (**B**).

In the fasting state, plasma NAA levels were numerically lower in the individuals with obesity compared to lean controls; however, this difference did not reach statistical significance. From baseline to steady state, insulin levels increased from a mean of 66 to 447 pmol/L (*p* < 0.0001). During this period, mean circulating NAA concentrations declined slightly by 5% (fasting: 68.4 [64.7–77.6] vs. insulin clamp: 65.0 [57.0–75.3] ng/mL, *p* = 0.01), with a similar change observed in both groups. As shown in Figure 1, the decrease in NAA levels was not consistent across all subjects.

### 2.2. Associations Between ssNAA Levels with Metabolic Markers

**Figure 2:**
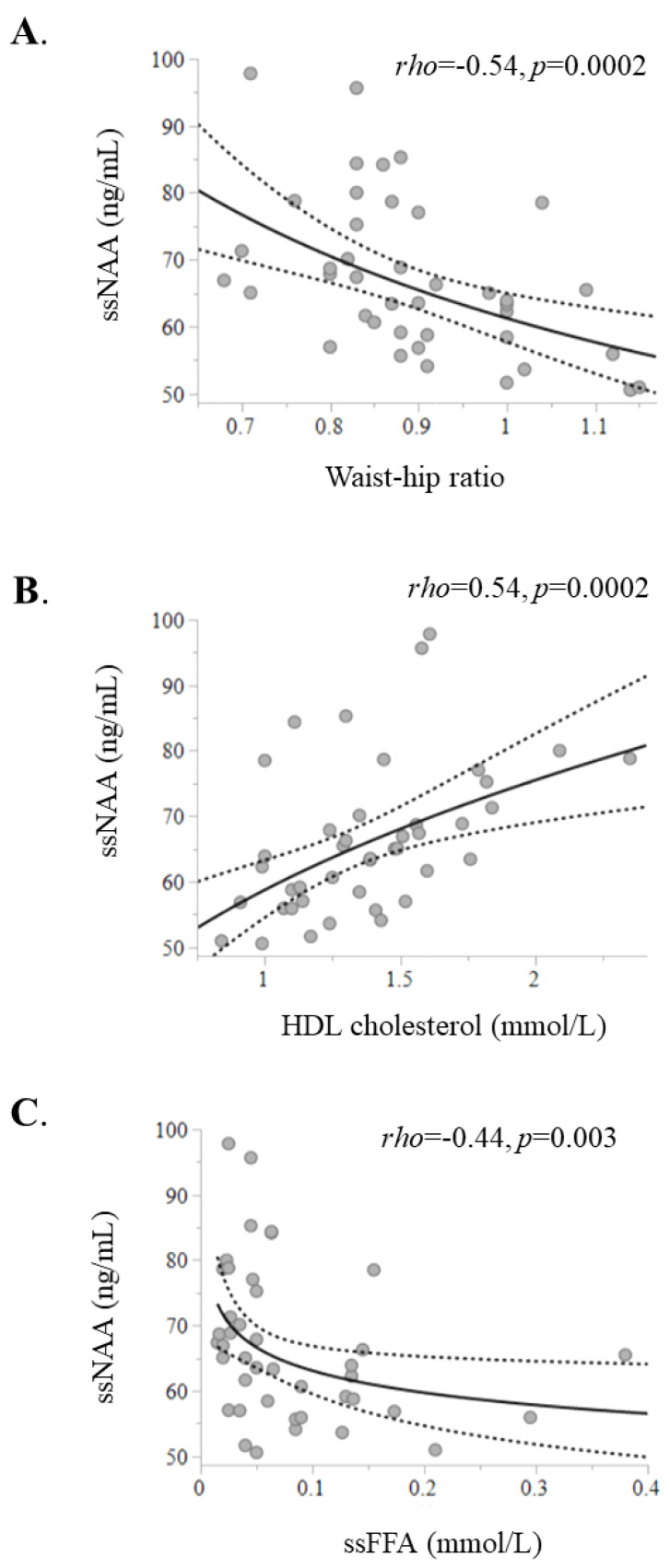
Circulating NAA correlates inversely with waist–hip ratio (**A**) and steady-state FFA (**C**) and directly with high-density lipoprotein (HDL) cholesterol levels (**B**).

**Figure 3:**
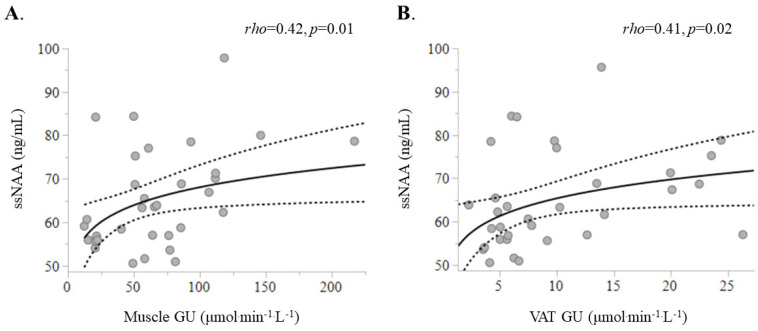
Circulating NAA correlates directly with skeletal muscle (**A**) and visceral adipose tissue (**B**) glucose uptake rates.

Plasma ssNAA levels were inversely associated with waist–hip ratio (*rho* = −0.54, *p* = 0.0002) and ssFFA (*rho* = −0.44, *p* = 0.003) and directly associated with high-density lipoprotein (HDL) cholesterol (*rho* = 0.54, *p* = 0.0002) (Figure 2) and serum adiponectin (*rho* = 0.48, *p* = 0.003). The ssNAA levels were directly related to whole-body insulin sensitivity (indexed by the M value) (*rho* = 0.34, *p* = 0.03). Additionally, they showed a positive correlation with skeletal muscle GU (*rho* = 0.42, *p* = 0.01) and VAT GU (*rho* = 0.41, *p* = 0.02) (Figure 3). Conversely, ssNAA levels were not directly associated with SAT GU (*p* = 0.3) or brain GU (*p* = 0.4). Most of these associations were confirmed when fasting NAA levels were used, albeit with weaker correlations (Table 2). This pattern of correlations was also confirmed in sensitivity analysis after excluding the four patients with T2D (Supplementary Data, Supplementary Table S1).

The change in plasma NAA levels from fasting to the insulin clamp state was inversely correlated with the change in plasma acetate levels (*r* = −0.36, *p* = 0.048) (Figure 1).

## 3. Discussion

The present study yielded two main findings: First, in neurologically healthy patients across a wide range of body mass indexes (BMIs), acute exposure to supraphysiologic systemic insulin levels led to a small reduction in circulating NAA levels. This decrease in NAA levels was inversely related to the concomitant change in plasma acetate levels. Second, circulating NAA levels measured during euglycemic hyperinsulinemic conditions were associated with the classical markers of systemic insulin sensitivity as well as tissue-specific insulin sensitivity of skeletal muscle and visceral adipose tissue.

NAA is the second most abundant metabolite of the human brain, and its study has predominantly focused on neurological disorders for several decades. Early reports indicated that NAA does not cross the blood–brain barrier [15]; however, patients with excessive brain NAA levels also exhibited increased NAA levels in the blood and urine [16], suggesting that brain NAA might spill over into the systemic circulation. Subsequently, it was discovered that N-acetyltransferase 8-like (*Nat8l*), the gene encoding aspartate N-acetyltransferase (the enzyme responsible for producing NAA from acetyl-CoA and aspartate), is expressed in peripheral tissues, with particularly high expression levels in brown adipose tissue (BAT) and, to a lesser extent, also in white adipose tissue [17]. Additionally, NAA was detected in quantifiable—albeit minor—concentrations in several common foods [18], indicating that circulating NAA levels might be derived from the diet. In this study, we could not determine the origin of circulating NAA.

To date, only a few preclinical studies have evaluated NAA metabolism (secretion and degradation) in peripheral tissues. It was shown that *Nat8l* expression is significantly decreased in white and brown adipose tissues in ob/ob mice, which remains unchanged in the brain compared to wild-type mice [17]. In genetically induced diabetic mice, ASPA activity in the duodenum is enhanced compared to control mice, suggesting some involvement in the pathophysiology of diabetic autonomic neuropathy [19]. Although the roles of NAA in the brain and periphery are not well elucidated, researchers proposed that both central and peripheral NAA could serve as storage and transport forms of acetate. Acetate could then be utilized for the synthesis of acetyl-CoA by acetyl-CoA synthase-1 when needed [20,21]. The current results in humans in vivo are in line with this notion, as we found that the change in plasma NAA levels was inversely related to the change in plasma acetate concentrations in paired fasting and insulin clamp experiments.

Moreover, although the expression of *Nat8l* in SAT or VAT biopsies was not assessed in the present study, plasma NAA levels were numerically lower in individuals with obesity compared to lean controls. A significant inverse association between circulating NAA and BMI was observed in a previous relatively large dataset from our group [10]. Moreover, that study demonstrated that, independent of age and BMI, plasma NAA levels were inversely related to hemoglobin A1c (HbA_1c_) and fasting plasma glucose levels [10]. Consistently, in a subset of patients with repeated circulating NAA measurements following either bariatric surgery or anti-diabetic treatment, plasma NAA levels increased [10]. In this study, we extended previous findings by showing that plasma NAA levels were directly related with whole-body insulin sensitivity, skeletal muscle GU, and visceral adipose tissue GU.

Skeletal muscle accounts for the majority of glucose disposal after meal ingestion or during hyperinsulinemia [22]. VAT contributes significantly less to glucose disposal, accounting for only about 5–10% of whole-body glucose uptake [23]. In both tissues, a higher GU indicates better tissue-specific insulin sensitivity [22]. VAT is considered a more metabolically active tissue compared to SAT. In the present dataset, we did not detect any association between plasma NAA and SAT GU—not even a trend—suggesting that NAA may indeed be more related to visceral AT metabolism. However, given the relatively small sample size, the lack of association between SAT GU and circulating NAA could be attributed to a type 2 statistical error. Circulating NAA was also not related to brain GU; this observation aligns with our previous study, which found no evidence of circulating NAA being associated with central NAA, small vessel cerebral disease, or cognitive function [14]. This suggests that circulating NAA may signal peripheral, but not central, phenomena.

In a recent study, Felix and colleagues elegantly developed an *ASPA* KO mouse model [24]. Through a series of experiments, they demonstrated that NAA accumulation in the white adipose tissue of KO animals stimulated the pentose phosphate pathway and pyrimide production. As glucose serves as the main carbon precursor for biosynthetic processes, the authors suggested that NAA accumulation in white adipose tissue “directs the prioritized flux of carbon into pyrimidine synthesis”. Of important note, using data from the Atherosclerosis Risk In Communities (ARIC) study, they also demonstrated an association between circulating NAA levels and the pyrimidine intermediate orotidine in humans, with obesity modulating this relationship. Taken together, these findings provide a potential mechanistic explanation for the direct association between circulating NAA and VAT GU in our study. Specifically, we speculated that higher circulating NAA levels may lead to increased intra-adipocyte NAA accumulation, which in turn enhances glucose uptake—potentially to support the pentose phosphate pathway.

One of the strengths of the present study is the thorough characterization of the study participants under two metabolic conditions. State-of-the art methods were employed, including the euglycemic hyperinsulinemic clamp technique to assess whole-body insulin sensitivity, metabolic imaging to evaluate tissue-specific insulin sensitivity, and NMR metabolomics performed both in fasting and ~120 min into the insulin clamp. Moreover, circulating NAA was measured with HPLC-MS-MS, a method providing high sensitivity, reproducibility, and accuracy, compared to other methods [25]. However, our study also has limitations. First and foremost, the data presented here are primarily correlative. As with all observational studies, these correlations do not allow for definitive causal inferences regarding NAA metabolism and insulin resistance nor do they elucidate potential mechanistic links between NAA and insulin sensitivity. Given the limited data on circulating NAA levels in humans, we believe our findings may help guide future research into the potential roles of NAA in human metabolism. Second, although SAT and VAT samples were obtained, we did not assess adipose tissue ASPA activity and *Nat8l* expression due to technical reasons. Moreover, while previous preclinical studies identified BAT as an important site of NAA production, BAT scanning was not performed in this study. Future studies could investigate the effect of cold exposure—an intervention that activates BAT—on circulating NAA levels and determine whether plasma NAA correlates with measures of BAT function, such as BAT GU, perfusion, radiodensity, and UCP-1 expression. Finally, while minor amounts of NAA might be obtained from the diet, the dietary habits of the study participants were not assessed. Whether dietary NAA influences plasma NAA levels remains unclear, warranting further investigation.

In summary, we demonstrated that plasma NAA levels were affected by insulin and directly associated with markers of peripheral insulin sensitivity. While plasma NAA may be relevant to aspects of whole-body homeostasis, mechanistic insights are currently lacking. Further investigations are needed to elucidate the role of circulating NAA in peripheral and tissue-specific metabolism, a process that is beginning to be uncovered.

## 4. Materials and Methods

Participants and study design: A total of 24 persons living with obesity and 19 age- and sex-matched healthy, lean controls were studied. Persons with obesity were candidates for metabolic bariatric surgery and were consecutively recruited from the Division of Digestive Surgery of Turku University Hospital, Turku, Finland. Controls were recruited via advertisements in the local newspapers.

The inclusion and exclusion criteria were previously described [26]. In brief, subjects with obesity and lean subjects aged 18–60 years were recruited. All study participants were free of previous neurological disease (e.g., cognitive impairment, large vessel stroke, seizure disorder, Parkinson’s disease, clinically significant traumatic brain injury, multiple sclerosis, or previous brain infection/meningitis), and none of them had any major psychiatric illnesses (e.g., schizophrenia, bipolar disorder) or reported substance abuse. All study participants underwent a screening visit where written, informed consent was provided before participating in the study and information regarding medical history and drug use was collected. Blood pressure was measured three times with an OMRON 711 automatic blood pressure monitor (Omron Corporate, Kyoto, Japan) in subjects seated for at least 10 min, and the average of the last two measurements was recorded. Fasting blood and urine samples were collected and a standard oral glucose tolerance test (75-g, OGTT) with frequent blood sampling every 30 min was performed. According to American Diabetes Association criteria [27], four persons with obesity had T2D; of them, one was on metformin and three were on a combination of metformin and SGLT2 inhibitors. All anti-diabetic drugs were suspended 3 days prior to the metabolic studies. The study protocol was approved by the Ethics Committee of the Hospital District of Southwestern Finland (ClinicalTrials.gov: NCT04343469: https://clinicaltrials.gov/study/NCT04343469.

Study protocol: After an overnight fast (10–12 h), study participants arrived at the facilities of the Turku PET Centre, Turku, Finland. After voiding the bladder, two catheters were inserted in the antecubital veins of each arm, one for the administration of a radiolabelled tracer, glucose, and insulin, and the other for arterialized blood sampling; a euglycemic hyperinsulinemic clamp was started, as previously described [28]. In brief, a primed-continuous insulin infusion was given at a rate of 40 mU·m^−2^·min^−1^, followed by a variable 20% dextrose infusion. Blood samples were drawn every 5, 30, and 60 min for the determination of plasma glucose, insulin, and free fatty acids, respectively. Sixty min into the clamp, subjects were positioned inside the PET scanner, 181 ± 9 MBq of [^18^F]FDG was injected, and radioactivity acquisition from the brain and abdominal and femoral regions was done. During the scan, the arm used for venous blood sampling was warmed with a heating pillow throughout the clamp study to obtain arterialized venous blood every 5 min for the determination of plasma radioactivity, as in previous studies [29,30]. Plasma and urinary radioactivity concentrations were measured using an automatic γ-counter (Wizard 1480; Wallac, Turku, Finland).

Quantification of brain glucose uptake: The method to quantify brain glucose uptake was previously reported [13]. In brief, the fractional uptake rate (FUR), the quotient of tissue activity divided by the integral of plasma activity from injection until the middle of the selected frame, was calculated for each voxel separately, as previously described [13]. Next, voxelwise GU (mmol/kg/min) was calculated using the formula GU = (FUR*Cp)/(LC*tissue density)*100, where Cp is the average plasma glucose concentration from the injection until the end of the brain scan, and LC is the lumped constant for the brain (set at 0.65).

Skeletal muscle, subcutaneous, and visceral PET data analysis: PET images were reconstructed in a 256 × 256 matrix after correction for decay time, dead time, and photon attenuation. Image analysis was performed using Carimas v.2.9 (http://www.turkupetcentre.fi/). To obtain the time–radioactivity curves, 7–10 consecutive regions of interest (ROIs) were manually drawn on PET/CT fusion images in the anterior femoral muscles, subcutaneous abdominal tissue (SAT), and visceral adipose tissue (VAT) (Supplementary Figure S1). Glucose metabolism was assessed using the fractional uptake rate (FUR); to obtain glucose uptake (GU) rates, FUR (1/min) was multiplied by plasma glucose concentration. GU rates are expressed in µmol/min per 100 mL of tissue.

Calculations: Insulin-stimulated glucose disposal (M value) was used as a measure of whole-body insulin sensitivity, as previously described [31]. The M value was calculated using the glucose infusion rates during the second hour of the clamp as a mean of three 20 min intervals. Estimated glomerular filtration rate (eGFR) was calculated by the Chronic Kidney Disease Epidemiology Collaboration (CKD-EPI) equation.

Plasma NAA measurement: Plasma NAA concentrations were assessed using fasting samples and samples obtained ~120 min after the start of the clamp protocol. The concentrations were measured by high-performance liquid chromatography coupled with tandem mass spectrometry (HPLC-MS-MS) and sample derivatization. This method, which was validated in compliance with EMA guidelines, was previously described in detail by Campi et al. [25].

Biochemical analyses: Plasma glucose was determined in duplicate by the glucose oxidase method (Analox GM9, Analox Instruments, London, UK). Serum insulin was measured by an automatized electro-chemiluminescence immunoassay (Cobas e601, Roche Diagnostics GmbH, Mannheim, Germany). Serum steady-state free fatty acids (ssFFA) were measured using an enzymatic assay (ACS-ACOD, Wako Chemicals GmbH, Neuss, Germany) on a Cobas c702 automatic analyser (Roche Diagnostics GmbH). Serum adiponectin was measured using HADCYMAG-61K panel (Merck/Millipore, Darmstadt, Germany).

Serum nuclear magnetic resonance (NMR) metabolomics: Serum metabolic biomarkers were quantified during the insulin clamp (~120 min from the start) using high-throughput proton NMR metabolomics (Nightingale Health Ltd., Helsinki, Finland). This method provides simultaneous quantification of routine lipids, fatty acid composition, and various low-molecular metabolites including amino acids, ketone bodies, and gluconeogenesis-related metabolites in molar concentration units and lipoprotein subclass profiling with lipid concentrations within 14 subclasses. The NMR metabolomics platform was described previously [32].

Statistical analysis: Continuous variables are summarized as mean ± SD or median [interquartile range] if not normally distributed; normality of distribution was assessed using the Shapiro–Wilk test. Between-groups’ comparisons were performed by *t*-test or Mann–Whitney U test, as appropriate. Correlations between circulating NAA and other parameters of interest were performed using Pearson’s correlation coefficient, or Spearman’s *rho*. Repeated measures ANOVA was used to analyse changes in plasma NAA concentrations across the two groups (patients with obesity and healthy, lean controls) under the two different conditions (fasting and insulin clamp). The sample size for this study was determined based on a power calculation for its primary outcome: assessing the correlation between insulin sensitivity and brain GU [29]. A post hoc power analysis for the present study indicated that, with α set at 0.05, a total sample size of 42, and effect size of 0.41 to 0.50, we had sufficient power (0.81 to 0.95) to assess correlations between circulating NAA and markers of adiposity or insulin resistance. A *p* < 0.05 was considered significant. Analyses were performed using JMP version 13.0 (SAS Institute, Cary, NC, USA). Power calculation was performed using G*Power (version 3.1.9.7).

## Figures and Tables

**Table 1:** Anthropometric and biochemical characteristics of the study participants ^o^.

	Patients with Obesity	Lean Controls	*p* Value
M/F	5/19	5/14	0.9
Age (years)	46 [40–55]	48 [34–53]	0.7
NGT/IFG&IGT/T2D	10/10/4	16/3/0	0.02
BMI (kg/m^2^)	39.5 [37.1–43.6]	22.9 [21.5–24.5]	<0.0001
W/H	0.91 [0.87–1.04]	0.83 [0.76–0.87]	0.0002
Total cholesterol (mmol/L)	4.4 ± 0.8	4.4 ± 0.9	>0.9
HDL cholesterol (mmol/L)	1.2 ± 0.2	1.6 ± 0.3	0.0006
Triglycerides (mmol/L)	1.2 [1.1–1.6]	0.8 [0.6–1.0]	0.0002
HbA_1c_ (mmol/mol)	36 [33–39]	34 [32–36]	0.04
M value (μmol·min^−1^·kg^−1^)	15.4 [9.9–21.4]	46.5 [34.4–53.6]	<0.0001
C-reactive protein (mg/L)	3.1 [2.0–4.8]	0.4 [0.2–0.8]	<0.0001
Serum adiponectin (ng/mL)	51.1 [35.6–65.5]	55.2 [28.5–92.2]	0.8
Fasting NAA (ng/mL)	67.6 [60.3–73.4]	71.6 [66.8–83.5]	0.05
ssNAA (ng/mL)	62.0 [55.9–74.8]	67.3 [62.3–75.3]	0.07
Brain GU * (μmol·min^−1^·100mL^−1^)	30.2 [23.3–35.2]	28.3 [16.7–33.8]	0.1
Muscle GU * (μmol·min^−1^·L^−1^)	41.0 [17.4–64.3]	67.6 [48.0–94.4]	0.005
SAT GU * (μmol·min^−1^·L^−1^)	4.2 [3.1–5.7]	9.4 [6.5–13.2]	<0.0001
VAT GU * (μmol·min^−1^·L^−1^)	5.7 [4.2–7.5]	14.1 [9.3–22.8]	0.0001

^o^ Entries are mean [interquartile range]; * available in 14 lean and in 19 individuals with obesity. NGT: normal glucose tolerance, IFG: impaired fasting glucose, IGT: impaired glucose tolerance, T2D: type 2 diabetes, W/H: waist-to-hip ratio, ssNAA: steady-state N-acetyl aspartate, GU: glucose uptake, VAT: visceral adipose tissue, SAT: subcutaneous adipose tissue.

**Table 2:** Spearman’s *rho* between fasting NAA and ssNAA with parameters of interest ^o^.

	Fasting NAA	ssNAA
BMI	−0.24	−0.23
W/H	−0.37	−0.54
M value	0.22	0.34
ssFFA	−0.36	−0.44
HDL cholesterol	0.36	0.54
Adiponectin	0.40	0.48
Skel. muscle GU	0.31	0.42
VAT GU	0.21	0.41
SAT GU	0.03	0.20
Brain GU	−0.07	−0.14

^o^ W/H: waist-to-hip ratio, HDL: high-density lipoprotein, ssNAA: steady-state N-acetylaspartate, ssFFA: steady-state free fatty acids, GU: glucose uptake, VAT: visceral adipose tissue, SAT: subcutaneous adipose tissue.

## Data Availability

Some or all datasets generated during and/or analysed during the current study are not publicly available but are available from the corresponding author on reasonable request.

## Supplementary Materials

The following supporting information can be downloaded at https://www.mdpi.com/article/10.3390/ijms26115107/s1.
