# Supplementary material for: Circulating N-Acetylaspartate Levels Associate with Measures of Peripheral and Tissue-Specific Insulin Sensitivity
Source: Int J Mol Sci. 2025 May 26;26(11):5107. doi: 10.3390/ijms26115107 (PMC12154542; doi:10.3390/ijms26115107)
Supplement: Supplementary file 1 [file ijms-26-05107-s001.zip › ijms-3608526-supplementary.pdf]

Circulating N-Acetylaspartate Levels Associate with Measures of Peripheral and Tissue-Specific Insulin Sensitivity

Eleni Rebelos[1,2,3*], Miikka-Juhani Honka[1], Aino Latva-Rasku[1], Johan Rajander[4], Paulina Salminen[5,6], Ioanna A. Anastasiou[3], Dimitris Kounatidis[3], Nikolaos Tentolouris[3], Beatrice Campi[7], Angela Dardano[2], Giuseppe Daniele[2], Alessandro Saba[8], Ele Ferrannini[7], Pirjo Nuutila[1,9,10]

NAA and peripheral metabolism

[1] Turku PET Centre, University of Turku, Turku, Finland

[2] Department of Clinical and Experimental Medicine, University of Pisa, Pisa, Italy

[3] Diabetes Center, First Department of Propaedeutic and Internal Medicine, Medical School, National and Kapodistrian University of Athens, Laiko General Hospital , 11527 Athens, Greece;

[4] Turku PET Centre, Accelerator Laboratory, Åbo Akademi University, Turku, Finland

[5] Division of Digestive Surgery and Urology, Turku University Hospital, Turku, Finland

[6] Department of Department of Surgery, University of Turku, Turku, Finland

[7] CNR, Institute of Clinical Physiology, Pisa, Italy

[8] Laboratory of Clinical Pathology, St. Chiara University Hospital, Pisa, Italy

[9] InFLAMES Research Flagship, University of Turku, 20014 Turku, Finland

[10] Department of Endocrinology, Turku University Hospital, Turku, Finland

SUPPLEMENTARY MATERIAL

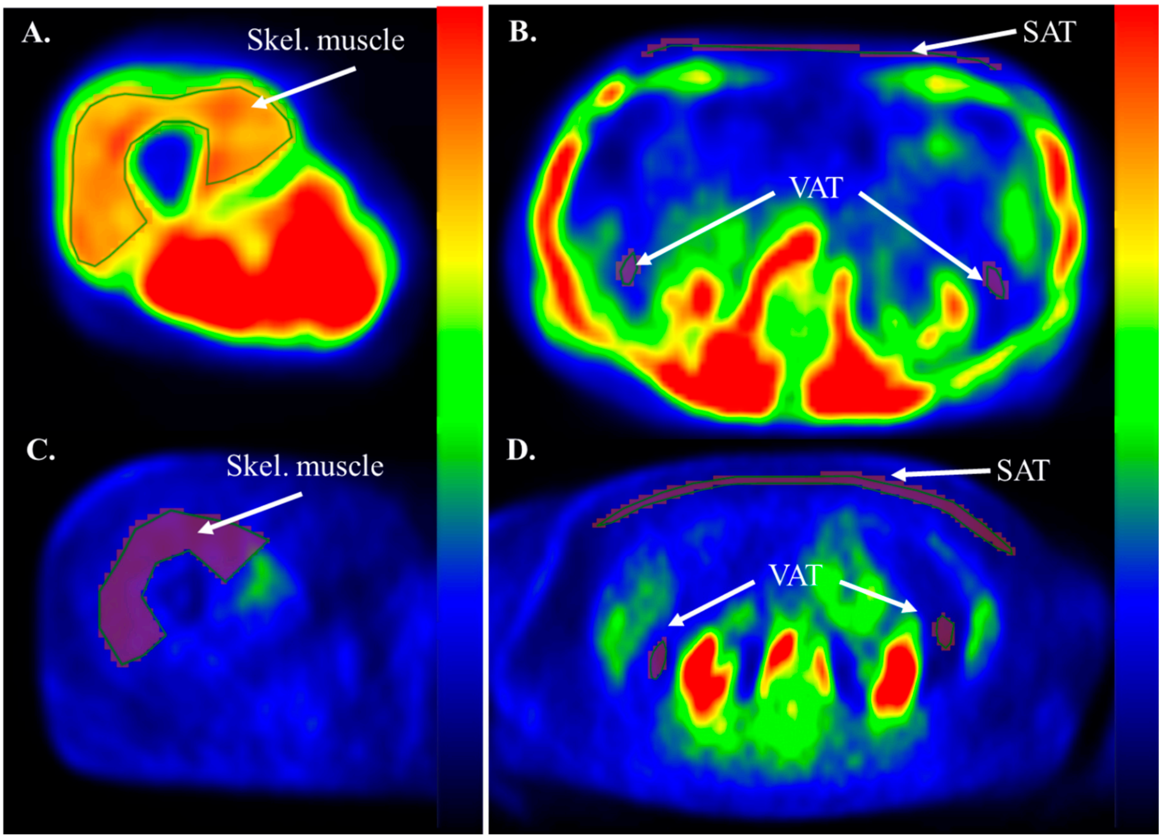

Supplementary Figure S1 – Illustration of region-of-interest placement for assessing glucose uptake in skeletal muscle, visceral adipose tissue (VAT) and subcutaneous (SAT) in [^{18}F]FDG-PET studies in a patient with obesity (A-B) and in a healthy lean control (C-D).

Supplementary Table S1- Spearman rho between fasting NAA and ssNAA with parameters of interest after excluding patients with type 2 diabetes.

	**Fasting NAA**		**ssNAA**	
	Rho	*p* value	*Rho*	*p* value
BMI	-0.30*	0.05	-0.22	0.2
W/H	-0.37*	0.01	-0.49	0.002
M value	0.30	0.06	0.30	0.06
ssFFA	-0.44	0.005	-0.42	0.008
HDL cholesterol	0.46	0.002	0.58	0.0001
Adiponectin	0.30	0.09	0.51	0.004
Skel. muscle GU	0.48	0.008	0.50	0.005
VAT GU	0.23	0.2	0.39	0.04
SAT GU	0.17	0.4	0.21	0.3
Brain GU	-0.10	0.5	-0.12	0.5
